# Actively Expressed Intergenic Genes Generated by Transposable Element Insertions in *Gossypium hirsutum* Cotton

**DOI:** 10.3390/plants13152079

**Published:** 2024-07-26

**Authors:** Yongzhuo Guan, Mingao Zhou, Congyu Zhang, Zixuan Han, Yinbao Zhang, Zhiguo Wu, Yuxian Zhu

**Affiliations:** 1 College of Life Sciences, Wuhan University, Wuhan 430072, China; 2 Xinjiang Jinfengyuan Seed Co., Ltd., Aksu City 843100, China; 3 Institute for Advanced Studies, Wuhan University, Wuhan 430072, China; 4 Hubei Hongshan Laboratory, Wuhan 430072, China; 5 TaiKang Center for Life and Medical Sciences, Wuhan University, Wuhan 430072, China

**Keywords:** *G. hirsutum*, ITGs, LTR insertion, histone modification, evolution

## Abstract

The genomes and annotated genes of allotetraploid cotton *Gossypium hirsutum* have been extensively studied in recent years. However, the expression, regulation, and evolution of intergenic genes (ITGs) have not been completely deciphered. In this study, we identified a novel set of actively expressed ITGs in *G. hirsutum* cotton, through transcriptome profiling based on deep sequencing data, as well as chromatin immunoprecipitation, followed by sequencing (ChIP-seq) of histone modifications and how the ITGs evolved. Totals of 17,567 and 8249 ITGs were identified in *G. hirsutum* and *Gossypium arboreum*, respectively. The expression of ITGs in *G. hirsutum* was significantly higher than that in *G. arboreum.* Moreover, longer exons were observed in *G. hirsutum* ITGs. Notably, 42.3% of the ITGs from *G. hirsutum* were generated by the long terminal repeat (LTR) insertions, while their proportion in genic genes was 19.9%. The H3K27ac and H3K4me3 modification proportions and intensities of ITGs were equivalent to genic genes. The H3K4me1 modifications were lower in ITGs. Additionally, evolution analyses revealed that the ITGs from *G. hirsutum* were mainly produced around 6.6 and 1.6 million years ago (Mya), later than the pegged time for genic genes, which is 7.0 Mya. The characterization of ITGs helps to elucidate the evolution of cotton genomes and shed more light on their biological functions in the transcriptional regulation of eukaryotic genes, along with the roles of histone modifications in speciation and diversification.

## 1. Introduction

Genes are parts of the DNA double helix. They possess and deliver a variety of specific genetic information by coding vital proteins that contribute to maintaining the basic structure and performance of organisms, through their roles in growth, development, decline, disease, aging, and death [1]. Intergenic genes (ITGs) are segments of DNA sequences and they are located between genes. Some ITGs affect or control gene performance, but most of them have no known function. Genomes of higher plants have more ITGs. For example, larger proportions of ITGs and more transposable elements explain why the genome of pine is extremely large [2]. Some studies reported that in maize, ITGs can evenly comprise ~85% of the whole genome [3].

Although an increasing number of whole genomes have been deciphered for plants, the molecular mechanism and functional characterization of ITGs still require much exploration. In fact, researchers are concerned about the high proportion of ITGs in plants. Various sequencing techniques, including third-generation sequencing [4], high-throughput transcriptome sequencing, and ChIP-seq [5,6] are applied in studying ITGs, through the joint analysis of multi-omics data. Genome-wide structural variation in maize was analyzed using PacBio RSII, a third-generation sequencing technology. This assisted in addressing the loss of large amounts of ITG information, which is usually experienced when short-read sequencing is employed [4]. Furthermore, ChIP-seq showed that in many plants, ITGs consisted of histone modifications, which possibly account for various heritable phenotypes [5,6].

The mechanism for the epigenetic dynamic regulation of genes in the ITGs of plants is still unclear. Histone modification is involved in precisely regulating target genes through histone variants and multiple modification combinations, thereby affecting the epigenetic mechanism of gene regulation [7,8]. H3K4me3, H3K27ac, and H3K4me1 are common types of histone modifications, which often coexist in a polyvalent modified pattern to enhance the precise regulation of targeted genes [9,10,11,12]. H3K4me3 is more concentrated at promoters that are near the transcription start sites (TSSs) of transcribing genes. It is regarded as an active transcriptional gene maker that promotes the binding of the transcription initiation complex before the process of transcription commences [13,14,15,16]. It has been reported that H3K27ac is often located at the promoter and enhancer regions. This weakens the binding ability of histone and nearby DNA by neutralizing the positive charge of the lysine residue via acetylation. Chromatin is kept loose and open, activating the transcription of genes [17]. When H3K27ac modification simultaneously occurs in this enhancer, the labeled gene is activated [18]. H3K4me1 largely modifies enhancers of labeled genes by recruiting complexes, some of which regulate chromatin, to adjust their activity [19].

Although many studies have discussed the distribution of ITGs, no systematic research on ITGs and associated histone modifications in *G. hirsutum* has been reported yet. In this study, the characteristics of genic genes and ITGs in tetraploid cotton (*G. hirsutum*) and diploid cotton (*G. arboreum*) were analyzed using transcriptome sequencing. Moreover, high-throughput sequencing (ChIP-Seq) was performed against the three histone modifications (H3K27ac, H3K4me3, and H3K4me1) in *G. hirsutum*, and their relationship with ITGs was comprehensively analyzed. Additionally, the origins of *G. hirsutum* were also determined based on the identification of genic and intergenic gene pairs [20]. 

## 2. Materials and Methods 

### 2.1. Plant Materials

*Gossypium hirsutum* L. (Xu142) was grown under controlled conditions of 15 h light at 32 °C and 9 h dark at 25 °C in a greenhouse at Wuhan University, Hubei, China. Tender leaves and zero-day post-anthesis (0 DPA) ovules were collected during the florescence period of the cotton plants for RNA extraction (TIANGEN, DP441, Beijing, China) and ChIP-seq. Three biological replicates were prepared for each sample to perform chromatin immunoprecipitation (ChIP)-seq analysis. 

### 2.2. Identification of ITGs and Exon Distributions of G. hirsutum and G. arboreum

In the identification of ITGs, 200G RNA-seq data of *G. hirsutum* (Xu142) was generated in this study, and 200G RNA-seq data of *G. arboreum* (Shixiya1) was downloaded from NCBI (PRJNA713422). These transcript data were mapped on the genomes *G. hirsutum* GCF_007990345.1 and *G. arboreum* GCF_025698485.1, respectively, using hisat2 (version 2.1.1) with the following parameters: hisat2-p 10-x Cottongenomedb-1 read1-2 read2-S Mappingresults.sam. The aligned results were assembled de novo by StringTie (version 2.2.3). The longest transcript for each gene was selected to calculate its expression. All samples were combined to obtain a non-redundant transcript set, which was then reassembled. Cuffcompare was used to divide the intergenic transcripts into two classes: those with a class code “u” to indicate that they were at least 1.5 kb away from a previously known gene and those with a class code “p,” which were 0.5–1.5 kb away from a known gene [6].

### 2.3. Transposable Element (TE) Insertion Analysis

Total cotton gene annotations and ITG annotations from the previous step, as well as the total cotton TE annotations from www.cottongen.org, were used to analyze the frequencies of insertion events. The insertion information between genic genes and ITGs was annotated using the “annotatePeaks.pl.” function of Hypergeometric Optimization of Motif EnRichment (HOMER) v4.11. The TE sequences that were within 1000 bp of the upstream or downstream regions, for genes and ITGs, were regarded as valid insertions. The insertion frequencies were then calculated based on the distances between TE and genic genes or ITGs.

### 2.4. ChIP-Seq Analysis of Histone Modifications on Genes and ITG Genes

Three histone modifications, which are H3K27ac (Abcam, Cambridge, UK, ab4729), H3K4me3 (Abcam, ab8580), and H3K4me1 (Abcam, ab8895), were used to analyze the regulatory mechanisms of genic genes and ITGs in cotton *Gossypium hirsutum* L. (Xu142). Chromatin immunoprecipitation, followed by the next-generation sequencing (ChIP-seq) method, was employed to estimate histone modification signal ratios and intensities as previously described (Yang et al. 2023 [6]). In brief, a 2 g mixture of *Gossypium hirsutum* L. (Xu142) tender leaf and 0 DPA ovule materials were harvested and cross-linked with 1% formaldehyde at room temperature for 30 min. The samples were then grounded in liquid nitrogen before being lysed in buffer 1 (0.4 M sucrose, 10 mM Tris-HCl (pH = 8.0), 5 mM β-Me, 1 × proteinase inhibitor cocktail, 2% PVPP). The nuclei were extracted using buffer 2 (0.25 M sucrose, 10 mM Tris-HCl (pH = 8.0), 5 mM β-Me, 10 mM MgCl_2_, 1% TritonX-100, 1 × proteinase inhibitor cocktail) and buffer 3 (1.7 M sucrose, 10 mM Tris-HCl (pH = 8.0), 5 mM β-Me, 2 mM MgCl_2_, 1% TritonX-100, 1 × proteinase inhibitor cocktail).

The nuclear chromatins were fragmented using a Covaris M220™ Focused ultrasonicator (Woburn, MA, USA) with SonoLab™7.2 (Nové Město, Czech Republic) at 14 W power, for 7 min, with the sonication buffer (1.7 M sucrose, 10 mM Tris-HCl (pH = 8.0), 5 mM β-Me, 2 mM MgCl_2_, 1% TritonX-100, 1 × proteinase inhibitor cocktail). The appropriate chromatin fragments with 300–500 bp were immunoprecipitated with 1 µg protein A/G-coupled antibodies against H3K27ac, H3K4me3, and H3K4me1 overnight, at 4 °C in RIPA-150 buffer (50 mM Tris-HCl pH 8.0, 150 mM NaCl, 1 mM EDTA, 1% Triton X-100, 0.1% NaDOC). The pellets were then sequentially washed with RIPA-150, RIPA-300 (50 mM Tris-HCl pH 8.0, 300 mM NaCl, 1 mM EDTA, 1% Triton X-100, 0.1% NaDOC), and RIPA-LiCl (50 mM Tris-HCl pH 8.0, 200 mM LiCl, 1 mM EDTA, 1% Triton X-100, 0.1% NaDOC) buffers, with each buffer being used two times. The pellets were digested by proteinase K at 37 °C for 1 h and 65 °C for 4 h. The DNA was purified using the TransGen Biotech kit (TransGen, EG101-01, Beijing, China). ChIP-seq libraries of the three histone modifications were prepared using the NEBNext^®^ Ultra™ II DNA Library Prep Kit (NEB #E7103, New England BioLabs, Ipswich, MA, USA). Each library was sequenced for 20 M reads using PE150 technology on the Illumina HiSeq 3000 platform (San Diego, CA, USA).

The nine histone modification clean data inputs (three replicates for each histone modification) and one clean data input were mapped on the upland cotton genome (*Ghirsutum*_TM-1_WHU_genome.standard.fa) using the Bowtie2 software (version 2.5.3). Peak calling was performed by HOMER packages to annotate histone modification peaks on chromosomes with default parameters. The histone modification peak signals in the +2 kb and −2 kb regions of the TSS of genes and ITGs were identified [21].

### 2.5. Evolution Analysis of Genic Genes and ITGs in G. hirsutum 

The evolution analysis strategies for genic genes and ITGs were derived from the GPDF method that was reported by Huang et al. 2020 [20]. In brief, the total genic genes and ITGs of *G. hirsutum* were scanned along with each of the annotated gene sequences using the BLASTN software (2.15.0). The E-value was set at 10^−5^. The identities between all homologous sequences were normalized to generate identity datasets. Identity curves between genic genes and ITGs were then obtained. The time taken for the genic genes and ITGs to emerge was calculated using the formula T = Ks/2r (Ks = 1 − (identity peak value)), where r is the nucleotide substitution rate and was set at 1.5 × 10^−8^.

### 2.6. Validation of Unique ITGs in G. hirsutum Using qRT-PCR Analysis

We mapped all ITG sequences from *G. hirsutum* on the database which was made of total ITGs from *G. arboreum* to determine the unique ITGs, which were analyzed by the BLASTN software. Then, we chose the ITGs with low sequence homology from *G. hirsutum* and *G. arboreum* and determined the RNA expression levels relative to H3. Leaves from *G.hirsutum* and *G. arboreum* (100 mg each) were separately extracted for RNA. The RNA was immediately reversed to cDNA for qRT-PCR analysis. We selected *Ghir_A02G014370.1* and *Garb_02G004400.1*, both of which are histone superfamily proteins, as the internal references for calculating the relative content of ITGs.

## 3. Results

### 3.1. A Significant Number of Actively Expressed ITGs Were Observed in G. hirsutum Cotton

**Figure 1:**
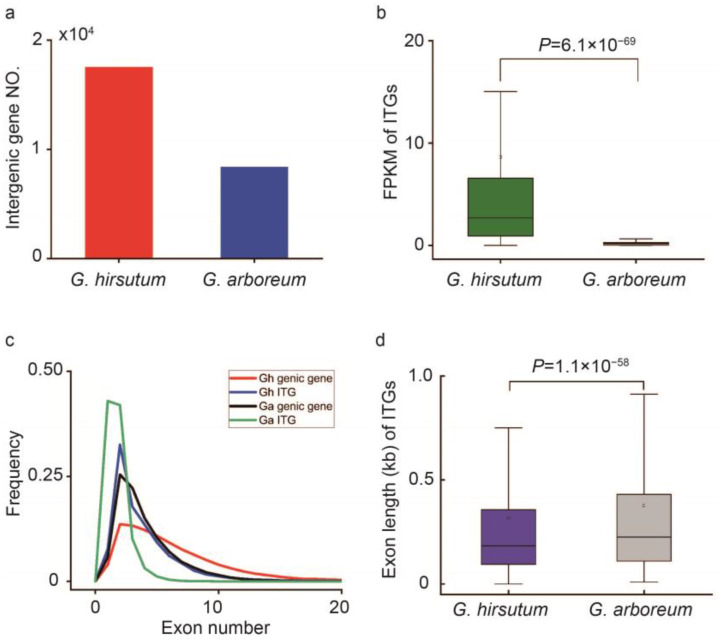
Characterization of the genic genes and ITGs obtained from tetraploid cotton *G. hirsutum* and diploid *G. arboreum*. (**a**) Histogram that shows the number (NO.) of ITGs from the transcriptome data of *G. hirsutum* and *G. arboreum*. (**b**) Boxplot that describes the FPKM of ITGs from *G. hirsutum* and *G. arboreum.* (**c**) Frequency curve chart that depicts the exon number distributions in genic genes and ITGs in *G. hirsutum* and *G. arboreum.* (**d**) Boxplot for the exon length distributions of ITGs in *G. hirsutum* and *G. arboreum.* One-Way ANOVA was used to analyze the significant difference of expression in *G. hirsutum* and *G. arboreum* (*p* < 0.01).

To explore the global features of *G. hirsutum and G. arboreum* transcriptomes, the genes were divided into two groups: genic genes and ITGs. The aligned reads for each sample were assembled de novo by StringTie, with some modifications as reported by Yang et al. (2023) [6]. A total of 17,567 and 8249 intergenic genes were identified in *G. hirsutum and G. arboretum,* respectively (Figure 1a). To further investigate the molecular mechanism for the formation of ITGs, the expression of ITGs in the two transcriptomes was analyzed. Interestingly, a significant difference in expression frequency was observed between *G. hirsutum and G. arboretum*. The Fragments Per Kilobase Million (FPKM) value for ITGs (medium = 2.81) was significantly higher in *G. hirsutum* than that in *G. arboreum* (medium = 0.086) (Figure 1b). Furthermore, the FPKM value of genic genes in *G. hirsutum* (medium = 5.15) was also higher than that in *G. arboreum* (medium = 2.03) (Figure S1a). Four unique ITGs were filtrated, and qRT-PCR was performed for *G. hirsutum* and *G. arboretum* (Table 1).

Statistical analysis of exon number frequency indicated that 7.61% of the ITGs in *G. hirsutum* were single-exon genes, 32.53% possessed two exons, while the others had more than two exons (Figure 1c). The proportion of ITGs with single exons and two exons was higher in *G. arboreum* (42.96% and 41.98%, respectively) than in *G. hirsutum* (Figure 1c). Based on these results, there is a higher probability that more multi-exons occurred throughout the genic region of *G. hirsutum* (Figure 1c). The exon length distribution of ITGs in the *G. hirsutum* and *G. arboreum* transcriptomes was analyzed and is shown in Figure 1d. Interestingly, the intergenic transcripts of *G. arboreum* (medium _(*G. arboreum*)_ = 376) had longer exons compared to those in *G. hirsutum* (medium _(*G. hirsutum*)_ = 315). This may be due to the higher proportion of single exons in *G. arboreum*. Similarly, the exon length of genic genes in *G. arboreum* was significantly longer than that of those in *G. hirsutum* (Figure S1b).

### 3.2. G. hirsutum ITGs Highly Correlated with the LTR TE Insertion Events

**Figure 2:**
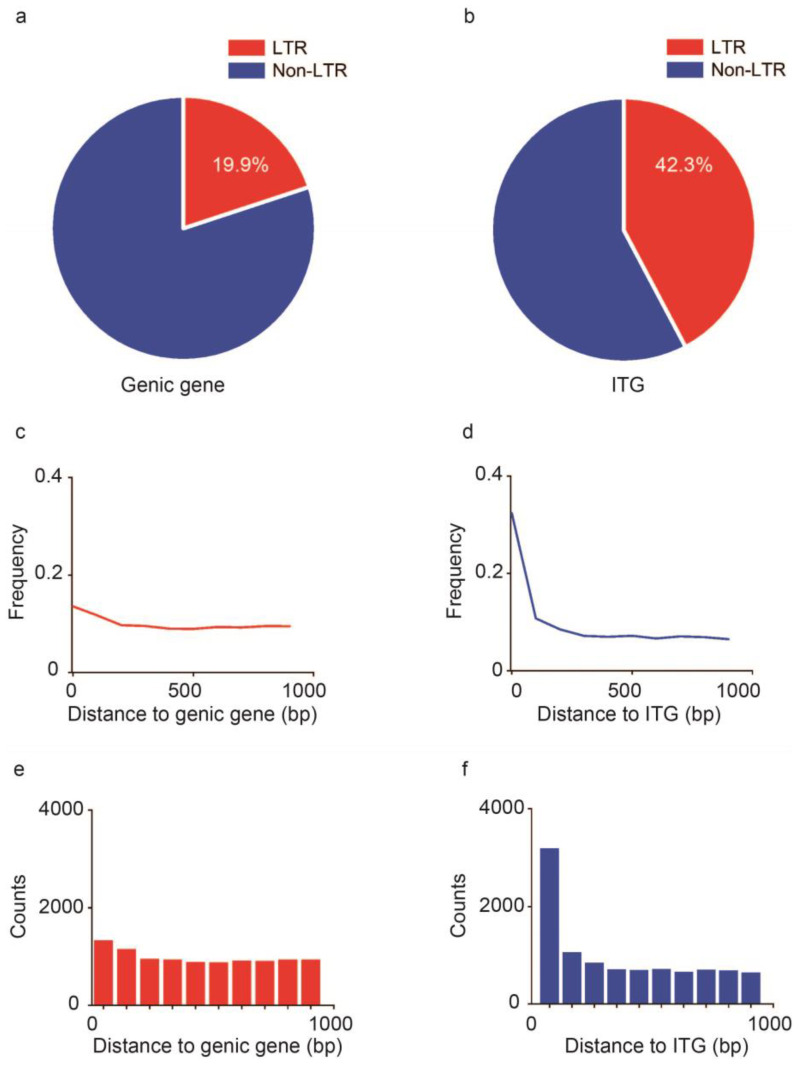
LTR insertion distributions in the regions of the genic genes and ITG are described. (**a**,**b**) Pie chart that shows the percentage of LTR insertion in the −1000 bp upstream and 1000 bp downstream regions of the genic genes and ITGs in *G. hirsutum.* (**c**,**d**) Frequency distributions of LTR insertion in the genic gene and ITG regions in *G. hirsutum* are displayed. (**e**,**f**) Count distributions of LTR insertions in the genic gene and ITG regions in *G. hirsutum*.

To further investigate the molecular mechanism for the formation of the ITGs, we analyzed the LTR frequency in the regions that were 1000 bp upstream and downstream along the genic genes and ITGs in *G. hirsutum*. A significant difference in the transposon insertion ratio was recorded between genic genes and ITGs. Only 19.9% of the genic genes contained LTRs in their exons (Figure 2a, Table 2). However, the transposon insertion rate was higher than 42.3% in ITGs (Figure 2b, Table 2). Notably, approximately 32.3% of the LTRs were within the range of less than 100 bp away from intergenic regions while only 13.6% occurred in the genic genes (Figure 2c,d).

There were subtle changes in LTR frequency along the 1000 bp range upstream and downstream of the genic regions. Therefore, a relatively stable range of 8.9–13.6% was maintained (Figure 2c). However, wider changes were observed in the LTR distribution frequency of ITGs, with a notable decrease from 32.3% to 7.2%, as the distance between LTRs and ITGs increased. The 7.2% value was recorded when the distance between LTRs and ITGs was greater than 500 bp. The LTR distribution frequency then stabilized around 7% (Figure 2d). Similarly, the distribution of LTR insertion counts in the region of genic genes and ITGs of *G. hirsutum* was also summarized (Figure 2e,f).

### 3.3. G. hirsutum ITGs Possessed Higher H3K4me1 Signal Proportion in Genic Genes Than That in ITG

**Figure 3:**
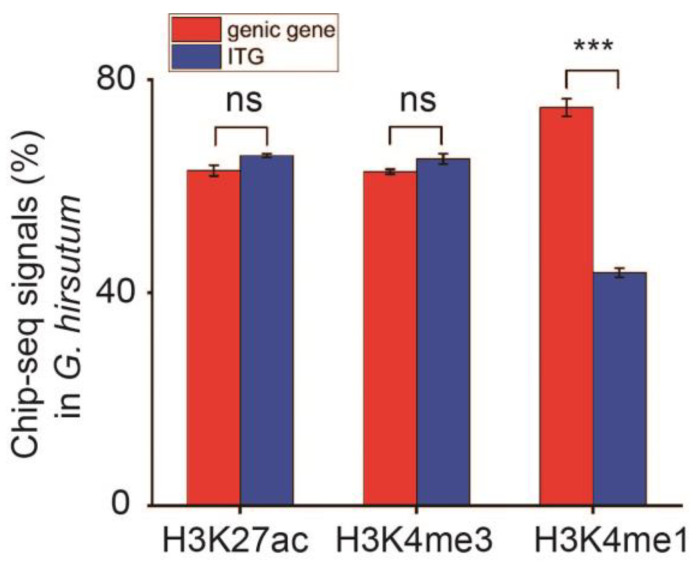
Overview of the three histone modifications in genic genes and ITGs in *G. hirsutum*. The global distribution feature of ChIP-seq signals in three histone modifications (H3K27ac, H3K4me3, and H3K4me1) on the genic gene and ITG levels. One-Way ANOVA was used to analyze the significant difference in ChIP-seq signal proportions of the three histone modifications between genic genes and ITGs (ns, not significant, *p* < 0.01, *** *p* < 0.001).

To explore the epigenetic dynamic regulation mechanism of genes in both genic and intergenic regions in *G. hirsutum*, ChIP-seq technology was employed, along with the global statistical analysis of three common histone modifications (H3K27ac, H3K4me3, and H3K4me1). ChIP-seq signals analysis showed the genic gene and ITG signal intensity of the three histone modification signals (Figure 3, Table 3). No significant differences were noted between genic gene and intergenic gene proportions related to H3K27ac and H3K4me3. However, the ratio of H3K27ac and H3K4me3 signals in ITGs was higher than that recorded for genic genes (Figure 3, Table 3). Otherwise, H3K4me3 signals were significantly more abundant in genic genes than in ITGs (Figure 3, Table 3). Additionally, the proportion for H3K4me1 signals was highest (74.8%) in the genic genes compared to H3K27ac (62.9%) and H3K4me3 (62.7%). On the other hand, the proportion for H3K4me1 in ITGs was the lowest when compared to the other two histone modifications (Figure 3, Table 3).

### 3.4. H3K27ac and H3K4me3 Modification Occupied an Important Position in G. hirsutum ITGs

**Figure 4:**
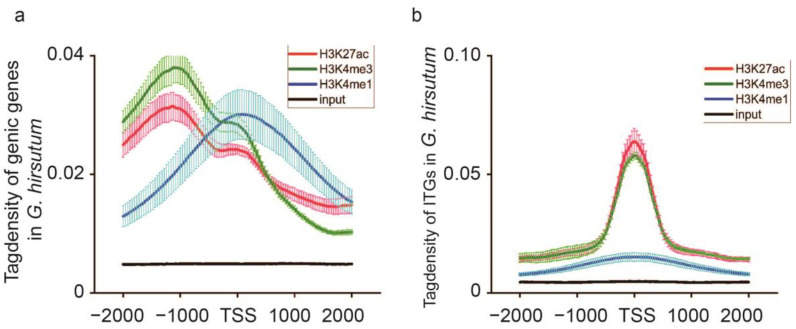
Tag density distribution of the three histone modifications. (**a**,**b**) Distribution of three histone modifications along genic genes and ITGs in *G. hirsutum.* A meta-gene profile was generated using normalized sequencing density, and the genic genes and ITGs were converted to proportions to normalize genes of different lengths. The 2 kb upstream and downstream regions of each gene were included.

The AnnotatePeals.pl script was used to annotate the enriched regions of the genic genes and ITGs that contained the three histone modifications. Genes were considered to have modifications when the signal occurred in the range within −2000 bp upstream and 2000 bp downstream. The intensity of enrichment in the three modifications occurring within genic and intergenic regions was statistically analyzed separately. The findings revealed that the H3K27ac and H3K4me3 signals were predominantly enriched in the genic regions, especially from −2000 bp upstream to the TSS site. The strongest enrichment intensity was reached within −1000 bp upstream (Figure 4a). Along the modified genic region with the extension in the 3′ direction, the signal was stronger, ranging from −2000 bp upstream to −1000 bp upstream. It then gradually decreased along the extension of the 3′ direction to the TSS site, before disappearing around 2000 bp downstream (Figure 4a). In contrast, H3K4me1 modifications were less distributed on the genic genes and mainly accumulated near the TSS site. Their distribution began to slowly increase from −2000 bp upstream to the TSS site with a maximum value and then gradually declined (Figure 4a).

H3K27ac and H3K4me3 signals that were located in the intergenic regions were enriched around the TSS site while the invariable H3K4me1 signal was recorded in the intergenic regions (Figure 4b). We concluded that the distribution characteristics of different types of histone modification signals were markedly different and this may be associated with their various independent mechanisms of regulating the activation of gene transcription. In conclusion, H3K27ac and H3K4me3 modifications have important roles in regulating the activation of gene transcription in both genic and intergenic regions.

### 3.5. G. hirsutum ITGs Were Generated 6.6 and 1.6 Mya

**Figure 5:**
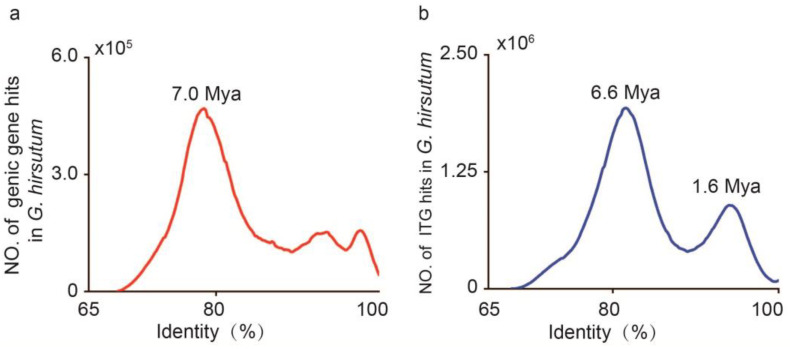
The origins of genic and intergenic transcripts from *G. hirsutum.* (**a**,**b**) Evolutionary analysis of the origins of genic genes and ITGs along *G. hirsutum* transcriptomes.

To further investigate the origins of genic genes and ITGs in *G. hirsutum*, we determined the identity of genic and intergenic genes from the plant’s transcripts. The evolution time of the major peaks was assessed using the Gaussian probability density function (GPDF), which was reported by Huang et al. [20]. One significant peak was discovered at identity of 78.6% (Figure 5a), which indicated that the genic genes from the At and Dt genomes in *G. hirsutum* were differentiated around 7.0 Mya (Figure 5a), while two distinct peaks with identity at 81.6% and 94.0% were observed in the ITGs of *G. hirsutum* (Figure 5b). The evolution time of the primary peak was estimated at 6.6 Mya, closely correlated with At and Dt genome differentiation time. Similarly, the secondary peak corresponded to 1.6 Mya, suggesting that the allotetraploid cotton may have generated around 1.6 Mya (Figure 5b).

## 4. Discussion

Whole genome assembly research showed that the content of ITGs is extremely high in plants, including maize [3], pine [2], spruce [22], and cotton [6]. Moreover, the functions of ITGs have attracted increasingly more attention from researchers. In this study, the number of ITGs was analyzed through de novo assembled transcripts in allotetraploid cotton *G. hirsutum* and diploid *G. arboreum.* The number of ITGs that were identified in allotetraploid cotton was almost double those in the diploid cotton. This reflected the differences in gene structure patterns in various species. Moreover, many of these ITGs were two-exon genes. However, *G. arboreum* possessed more single-exon genes [6]. On the other hand, *G. hirsutum* ITGs expressed higher FPKM compared to those in *G. arboretum*. This highlights the significance of ITGs, even in crucial biological functions. High proportions of LTRs in genomes were closely related to more intergenic transcripts, as confirmed in *G. arboreum* and *Z. mays* [6,20,23]. 

Our study was the first to discover a large number of ITGs in *G. hirsutum* derived from LTR insertions. The frequency distribution of LTR insertion in *G. hirsutum* genic regions was well proportioned. Higher proportions of LTRs were located less than 100 bp from ITGs and stabilized above 100 bp, which may be related to the stability and secrecy of ITGs. 

Further and systematic studies on the regulation of the expression of genic genes and ITGs in relation to transcriptional activation-related modifications (H3K27ac, H3K4me3, and H3K4me1) continued. In general, histone modification analysis is considered an auxiliary method for describing the transcriptional regulation of genes. According to ChIP-seq analysis against H3K27ac and H3K4me3, most of the genic genes and ITGs had transcription activation markers that were mainly located in the range from −2000 bp upstream to 2000 bp upstream. Similar reports mentioned that the majority of active histone markers against H3K27ac and H3K4me3 are mainly congregated at the promoter or the transcribed regions [6,24,25,26]. In cotton, H3K4me3 positively correlated with DNase I-hypersensitive site accessibility in promoter regions [27,28,29]. In this study, a large difference in H3K4me1 was observed between genic genes and ITGs in *G. hirsutum*, and this may affect the transcriptional activation of genes during the development of cotton. H3K4me1 specific binding protein was involved in RNA-mediated DNA methylation processes that regulate biological and abiotic stress, plant regeneration, plant growth, and fruit maturation processes [30]. 

Evolutionary studies with total genic genes have suggested that the At and Dt sub-genome was separated 7.0 Mya, which was earlier than the evolutionary time (6.6 Mya) of the primary peak of ITGs. The secondary peak of ITGs 1.6 Mya was probably correlated with the formation of allotetraploid cotton. Interestingly, Huang et al. (2020) [20] reported that the allotetraploid cotton may have formed as early as 2.0 Mya, through the calculation of LTR/TE insertion events. In this study, we suggest that ITGs probably evolve faster than genic genes. In summary, our study outlines the distribution characteristics of ITGs in *G. hirsutum*, especially their abundant number and active expression. The findings from this study contribute to the knowledge regarding genome evolution and diversification.

## 5. Conclusions

This study was the first to identify a large number of ITGs in *G. hirsutum* through transcriptome profiling based on deep sequencing data, which supplemented the annotated information of the *G. hirsutum* genome. Understanding the identified ITGs with higher expression than that in *G. arboreum* helps to elucidate the plant diversity and evolution of cotton genomes. When we executed the further investigation of the molecular mechanism for the formation of ITGs, we found that 42.3% of the ITGs from *G. hirsutum* were generated by LTRs. 

Most of the ITGs had H3K27ac and H3K4me3 modifications, which may affect the transcriptional activation of genes during the development of cotton. Evolution analyses with the ITGs from *G. hirsutum* have suggested that *Gossypium* were mainly produced around 6.6 and 1.6 Mya, later than the pegged time for genic genes, which is 7.0 Mya. In summary, our results suggest that evaluation of the characteristics of ITGs is a prerequisite for studying organisms, and they may have a significant impact during *Gossypium* genome evolution and diversification.

## Figures and Tables

**Table 1:** Relative expression of the four ITGs in *G. hirsutum* and *G. arboreum* at the RNA level.

Target	Species	Relative Expression to H3	STDEV
ITG1	*G. hirsutum*	0.023	0.00034
ITG2	*G. hirsutum*	0.0072	0.00038
ITG3	*G. hirsutum*	0.0038	0.000090
ITG4	*G. hirsutum*	0.0036	0.00030
ITG1	*G. arboreum*	0.000047	0.0000031
ITG2	*G. arboreum*	0	0
ITG3	*G. arboreum*	0.000010	0.00000069
ITG4	*G. arboreum*	0	0

**Table 2:** The percentage of LTR insertions in the regions of the genic genes and ITGs in *G. hirsutum*.

Gene Type	LTR Number	Total	LTR Percentage (%)
Genic genes	9841	49358	19.9
ITGs	7849	18576	42.3

**Table 3:** The percentage of the three histone modifications (H3K27ac, H3K4me3, and H3K4me1) on the genic gene and ITG levels.

Histone Modification	Gene Type	Repeat1 (%)	Repeat2 (%)	Repeat3 (%)	Mean (%)	STDEV
H3K27ac	genic genes	64.1	62.7	62.1	63.0	0.010
H3K27ac	ITGs	66.1	65.4	65.7	65.8	0.0035
H3K4me3	genic genes	62.8	62.3	63.1	62.7	0.0045
H3K4me3	ITGs	64.7	64.5	66.2	65.1	0.0095
H3K4me1	genic genes	75.6	75.9	72.9	74.8	0.016
H3K4me1	ITGs	44.5	42.8	44.8	43.8	0.0089

## Data Availability

Data are available in a publicly accessible repository. The data presented in this study are openly available from the National Center for Biotechnology Information under reference numbers PRJNA713422 (https://www.ncbi.nlm.nih.gov/search/all/?term=PRJNA713422 (accessed on 10 March 2021)), PRJNA1130147 (https://dataview.ncbi.nlm.nih.gov/object/PRJNA1130147 (accessed on 1 July 2024)), and PRJNA1130001 (https://dataview.ncbi.nlm.nih.gov/object/PRJNA1130001 (accessed on 30 June 2024)).

## Supplementary Materials

The following supporting information can be downloaded at https://www.mdpi.com/article/10.3390/plants13152079/s1, Table S1. List of ITG genes discovered from transcriptome analysis in *G. hirsutum*. Table S2. List of ITG genes discovered from transcriptome analysis in *G. arboreum*. Table S3. FPKM of genic genes and ITGs from *G. hirsutum* and *G. arboreum*. Table S4. List of the qRT-PCR primers used for the four unique ITGs and internal references in *G. hirsutum* and *G. arboreum*, and qRT-PCR results. Table S5. The exon number distributions of genic genes and ITGs in *G. hirsutum* and *G. arboreum*. Table S6. The exon length distributions of genic genes and ITGs in *G. hirsutum* and *G. arboreum*. Table S7. The location distributions of TEs in the genic and ITG regions in *G. hirsutum*. Tables S8–S10. The location and feature distributions of ChIP-seq peaks (H3K27ac, H3K4me3, and H3K4me1) on the genic gene and ITG levels in *G. hirsutum*. Table S11. Tag density distribution of H3K27ac, H3K4me3, and H3K4me1 in *G. hirsutum*. Table S12. The normalized identities between all homologous sequences originating from genic genes and ITGs in *G. hirsutum*. Figure S1. (a) Boxplot describing the FPKM of genic genes from *G. hirsutum* and *G. arboreum.* (b) Boxplot describing exon length distributions of genic genes in *G. hirsutum* and *G. arboreum.*
