# Supplementary material for: Actively Expressed Intergenic Genes Generated by Transposable Element Insertions in Gossypium hirsutum Cotton
Source: Plants (Basel). 2024 Jul 26;13(15):2079. doi: 10.3390/plants13152079 (PMC11314067; doi:10.3390/plants13152079)
Supplement: Supplementary file 1 [file plants-13-02079-s001.zip › Plants-Supplementary information.pdf]

Supplementary information

Actively expressed intergenic genes generated by TE insertions in *Gossypium hirsutum* cotton

Yongzhuo Guan[1], Mingao Zhou[1], Congyu Zhang[1], Zixuan Han[1], Yinbao Zhang[2], Zhiguo Wu[1*] and Yuxian Zhu[1,3,4,5]

1. College of Life Sciences, Wuhan University, Wuhan 430072, China

2. Xinjiang Jinfengyuan Seed Co., Ltd., Aksu City, Xinjiang, China

3. Institute for Advanced Studies, Wuhan University, Wuhan 430072, China

4. Hubei Hongshan Laboratory, Wuhan 430072, China

5. TaiKang Center for Life and Medical Sciences, Wuhan University, Wuhan 430072, China

*Correspondence: Zhiguo Wu, e-mail address: wu.zhiguo@whu.edu.cn (Z. Wu)

Table S1. List of ITG genes discovered from transcriptome analysis in *G. hirsutum*.

Table S2. List of ITG genes discovered from transcriptome analysis in *G. arboreum*

Table S3. FPKM of genic genes and ITGs from *G. hirsutum* and *G. arboreum*

Table S4. List of the qRT-PCR primers use for the four unique ITGs and internal references in *G. hirsutum* and *G. arboreum*, and qRT-PCR results

Table S5. The exon number distributions of genic genes and ITGs in *G. hirsutum* and *G. arboreum*

Table S6. The exon length distributions of genic genes and ITGs in *G. hirsutum* and *G. arboreum*

Table S7. The location distributions of TEs in the genic and ITG regions in *G. hirsutum*

Table S8-10. The location and feature distributions of ChIP-seq peaks (H3K27ac, H3K4me3, and H3K4me1) on the genic gene and ITG levels in *G. hirsutum*

Table S11. Tag density distribution of H3K27ac, H3K4me3, and H3K4me1 in *G. hirsutum*

Table S12. The normalized identities between all homologous sequences origined from genic gene and ITGs in *G. hirsutum*

Figure S1

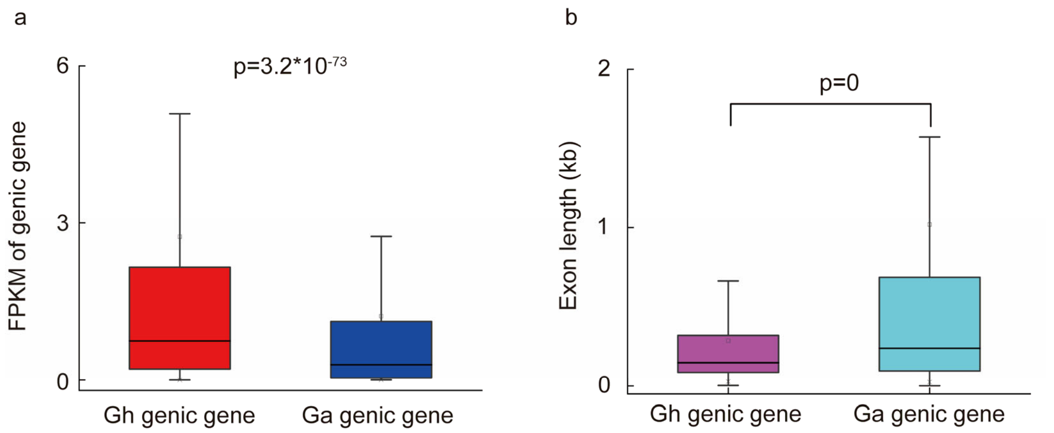

Figure S1. (a) Boxplot describing the FPKM of genic genes from *G. hirsutum* and *G. arboreum.* (b) Boxplot describing exon length distributions of genic genes in *G. hirsutum* and *G. arboreum.*
